# Tool-specific performance of vibration-reducing gloves for attenuating fingers-transmitted vibration

**DOI:** 10.3233/OER-160235

**Published:** 2016

**Authors:** Daniel E. Welcome, Ren G. Dong, Xueyan S. Xu, Christopher Warren, Thomas W. McDowell

**Affiliations:** Engineering & Control Technology Branch, National Institute for Occupational Safety and Health, Morgantown, WV, USA

**Keywords:** Anti-vibration glove, finger vibration, hand-arm vibration, hand-transmitted vibration, hand-arm vibration syndrome

## Abstract

**BACKGROUND:**

Fingers-transmitted vibration can cause vibration-induced white finger. The effectiveness of vibration-reducing (VR) gloves for reducing hand transmitted vibration to the fingers has not been sufficiently examined.

**OBJECTIVE:**

The objective of this study is to examine tool-specific performance of VR gloves for reducing finger-transmitted vibrations in three orthogonal directions (3D) from powered hand tools.

**METHODS:**

A transfer function method was used to estimate the tool-specific effectiveness of four typical VR gloves. The transfer functions of the VR glove fingers in three directions were either measured in this study or during a previous study using a 3D laser vibrometer. More than seventy vibration spectra of various tools or machines were used in the estimations.

**RESULTS:**

When assessed based on frequency-weighted acceleration, the gloves provided little vibration reduction. In some cases, the gloves amplified the vibration by more than 10%, especially the neoprene glove. However, the neoprene glove did the best when the assessment was based on unweighted acceleration. The neoprene glove was able to reduce the vibration by 10% or more of the unweighted vibration for 27 out of the 79 tools. If the dominant vibration of a tool handle or workpiece was in the shear direction relative to the fingers, as observed in the operation of needle scalers, hammer chisels, and bucking bars, the gloves did not reduce the vibration but increased it.

**CONCLUSIONS:**

This study confirmed that the effectiveness for reducing vibration varied with the gloves and the vibration reduction of each glove depended on tool, vibration direction to the fingers, and finger location. VR gloves, including certified anti-vibration gloves do not provide much vibration reduction when judged based on frequency-weighted acceleration. However, some of the VR gloves can provide more than 10% reduction of the unweighted vibration for some tools or workpieces. Tools and gloves can be matched for better effectiveness for protecting the fingers.

## 1. Introduction

Vibration-induced finger disorders, with vibration white finger their hallmark, are the major components of hand-arm vibration syndrome (HAVS) [1–4]. Hence, the major efforts for controlling HAVS are to reduce vibration-induced finger disorders. In principle, the most effective approach for control is to reduce the finger-transmitted vibration exposure. While this can be achieved through reducing tool handle vibrations and controlling exposure time, vibration-reducing (VR) gloves have also been proposed to help attenuate the vibration [5–7]. A laboratory study suggested that such gloves could reduce digital vascular responses to the vibration exposure [8]. An epidemiological study reported that such gloves effectively reduced some finger symptoms of HAVS at a workplace [9]. However, these gloves have not been officially considered as personal protection devices [10]. This is not only because there are few studies reporting their health effects, but also because it is unclear whether these gloves can effectively reduce the vibration transmitted to the fingers in the operations of powered hand tools.

The performance of VR gloves varies. To help differentiate and select them, the International Organization for Standardization (ISO) has set forth a test method [11,12]. The gloves that meet the criteria defined in the standard are classified as anti-vibration (AV) gloves, with the assumption that such gloves have better vibration-reducing performance than other gloves. Hence, AV gloves are a subset of VR gloves. Although finger vibration is of primary concern for controlling HAVS, the standard method does not use the measurement of the glove finger transmissibility at workplaces but rather it is based on the measurement of the glove transmissibility at the palm of the hand in a laboratory test [11]. This is because it is very difficult to reliably measure the vibration transmissibility of the glove fingers during tool operations at workplaces. For the same reasons, the vast majority of the reported studies investigated only the transmissibility of VR gloves at the palm of the hand [13–18], some of which indicate that VR gloves can reduce a portion of the vibrations transmitted to the palm in the operations of the tools with dominant vibrations above 25 Hz. Only a few laboratory studies examined the transmissibility of VR gloves at the fingers [19–21]. While a study suggested that certain work gloves could reduce the finger vibration at the proximal phalanges by 12–15% when used with one type of tool [22], a recent study estimated the effectiveness of an AV glove for reducing fingers-transmitted vibration on some tools [23]. Hence, the effectiveness of VR gloves for finger vibration reduction has not been investigated sufficiently.

Because there is lack of reliable information, the revised version of the standard [12] addresses finger protection by requiring that each AV glove must be a full-finger glove, the materials of the AV glove fingers are the same as those of glove palm, and the thickness of the glove fingers is greater than 0.55 times of that at the palm. These requirements can help specify the major mechanical properties of the gloves but they cannot guarantee the effectiveness of the glove fingers because the vibration attenuation depends not only on the glove mechanical properties but also the dynamic characteristics of the human fingers [24]. This theoretical prediction was verified in a recent study [21]. The reported transmissibility spectra suggest that the AV gloves are unlikely to substantially reduce the fingers-transmitted vibration and can amplify it throughout many of the frequency bands [21]. The glove effectiveness is also tool vibration-specific or vibration spectrum-specific. Without including the tool vibration spectrum in the analysis, it is not sufficient to prove the theoretical prediction.

The tool-specific performance of VR gloves can be estimated using a transfer function method [25–28], in which the vibration transmissibility spectra measured in a laboratory test and the tool vibration spectra measured at workplaces are used in the analysis. Many tool vibration spectra have been measured and reported [28] and the finger transmissibility spectra in three orthogonal directions using an advanced 3-D laser vibrometer are also available [21]. Therefore, the specific aims of this study are to further measure transmissibility spectra of additional gloves, to synthesize the representative spectra using the available data, and to estimate the tool-specific effectiveness of AV glove fingers for reducing human finger vibration exposure.

## 2. Methods

**Fig. 1:**
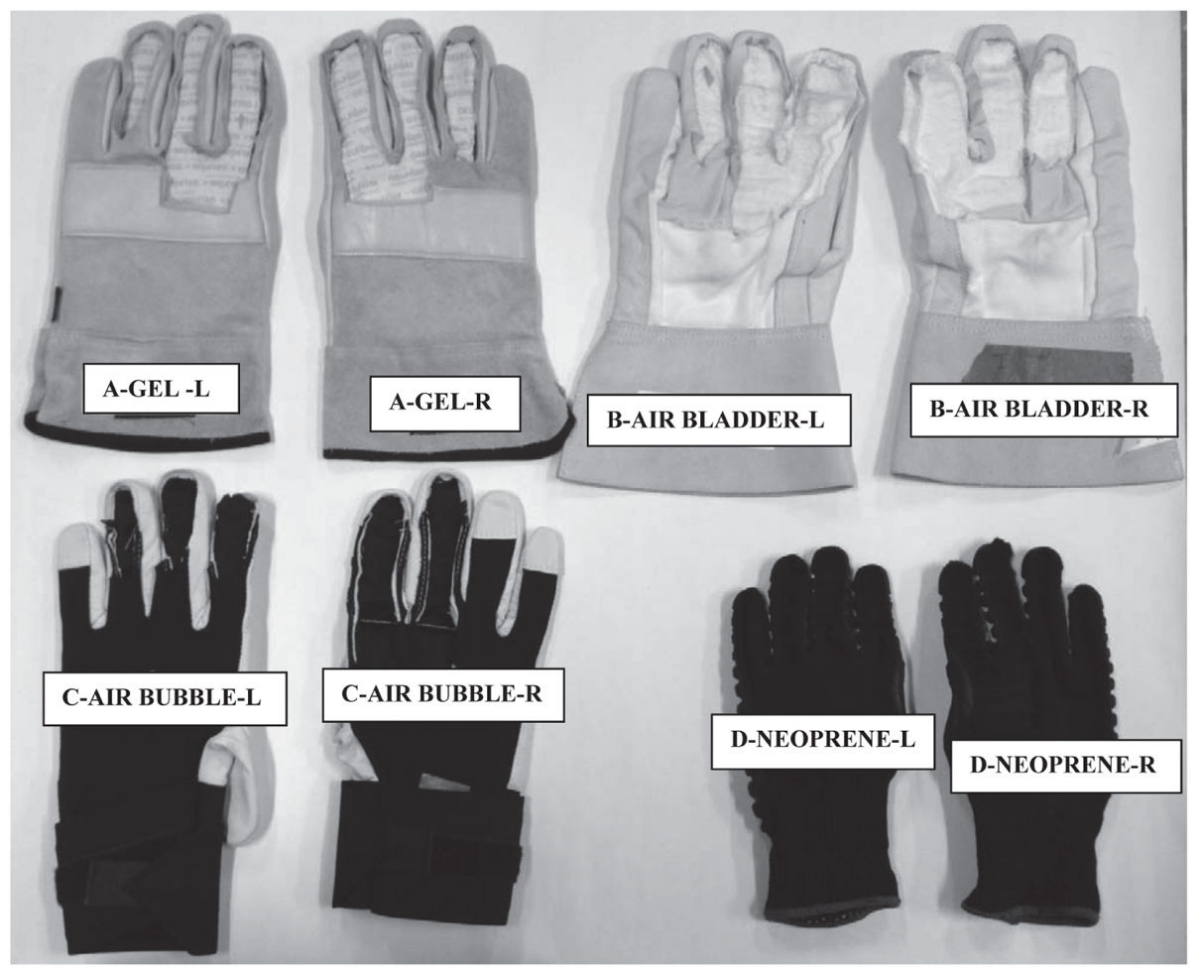
Four types of VR gloves (two for each type) considered in this study: Glove A – thick gel pad; Glove B – air bladder with pump; Glove C – cellular air bubbles; and Glove D – dipped neoprene.

The four VR gloves considered in this study are shown in Fig. 1. Their basic material characteristics, AV glove certifications, and grip strength influences are listed in Table 1. The transmissibility spectra at the fingers of two gloves (air bladder glove and gel-filled glove) were measured in a recent study [21]. The spectra of the other two gloves were measured in the current study.

### 2.1. Measurement of the vibration transmissibility spectra of gloves at the fingers

**Fig. 2:**
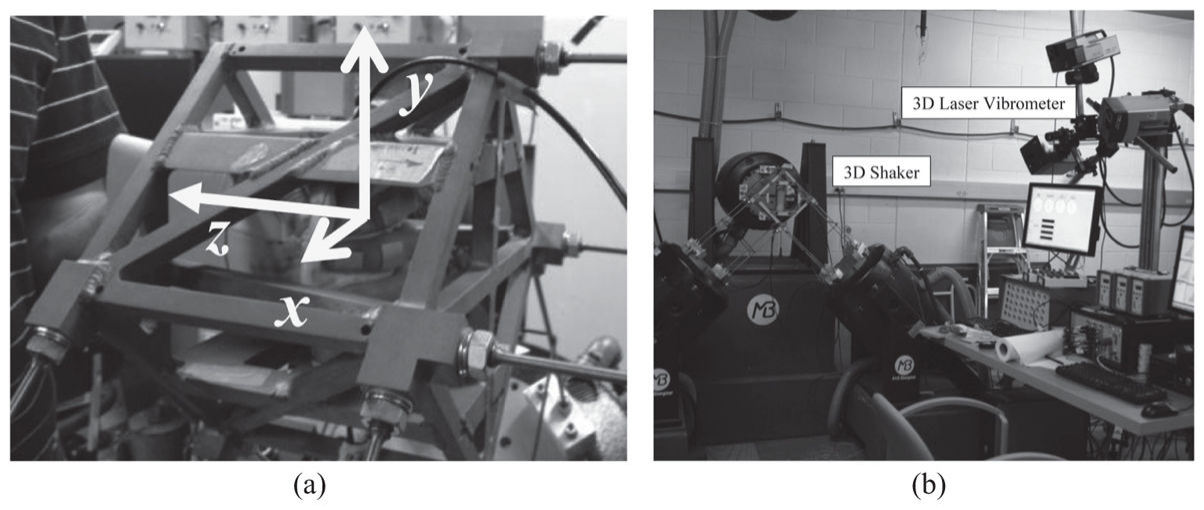
The test setup for the measurement of glove transmissibility spectra on the fingers in the three orthogonal directions (3-D: *x*, *y*, and *z*) using a 3-D laser vibrometer [21]. (a) a gloved hand on the instrumented handle. (b) the layout of the test equipment.

**Fig. 3:**
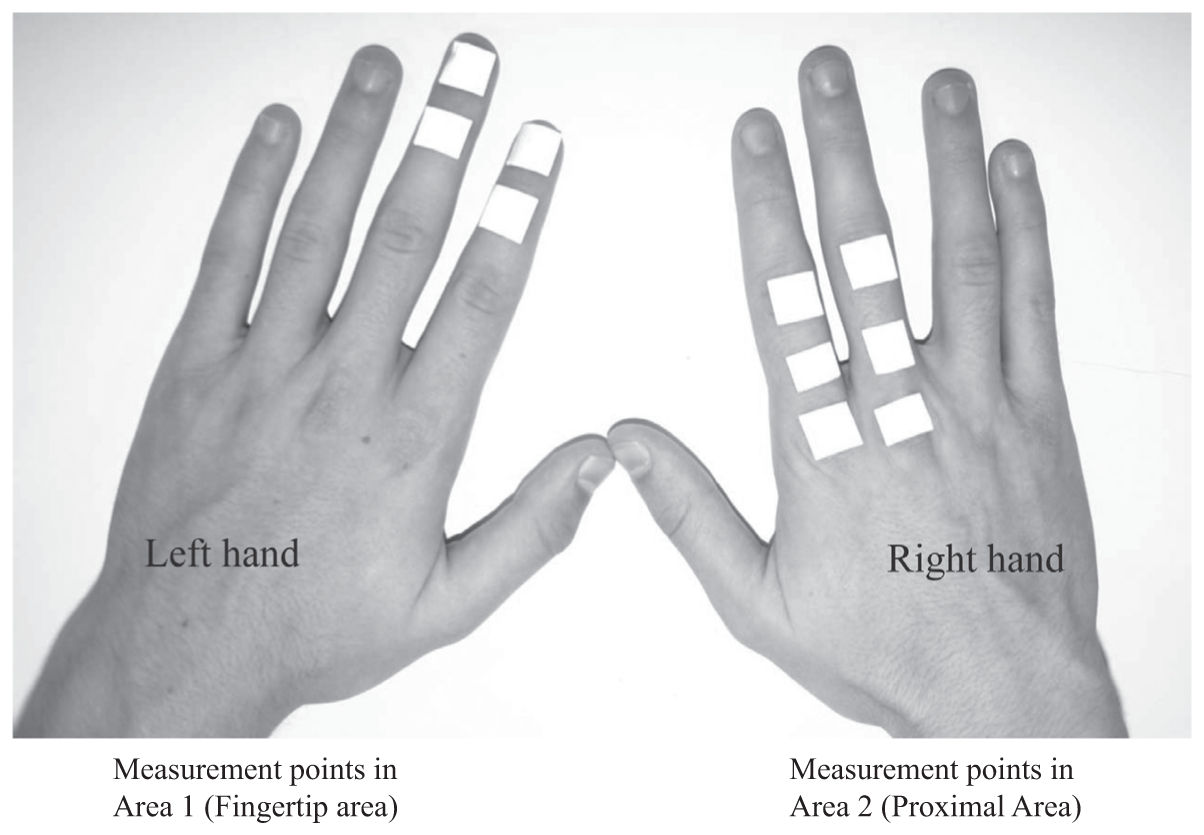
The points/locations in each of the two measurement areas: Area 1-fingernail, first knuckle, and middle phalangeal dorsum areas on the index and middle fingers of the left hand; Area 2-middle knuckle, proximal phalangeal dorsum, and third knuckle areas on the index and middle fingers of the right hand.

Six healthy male subjects participated in this experiment. Their anthropometric information is in Table 2. The present study used the same method as that used in our previous study [21]. Briefly, the measurement was carried out on a 3-D vibration test system (MB Dynamics, 3-D Hand-Arm Vibration Test System), as shown in Fig. 2. The multi-axis vibration controller of the 3-D hand-arm vibration test system was programmed to generate a broadband random vibration from 16 to 500 Hz in each of the three orthogonal directions (*x*, *y*, and *z*). The overall root-mean-square value of the acceleration in each direction was 19.6 m/s^2^. An instrumented handle equipped with a tri-axial accelerometer (Endevco, 65–100) and a pair of force sensors (Interface, SML-50) was used to measure the 3-D accelerations and applied grip force. Four hand forces (15 N grip, 30 N grip, 50 N grip, and combined 30 N grip and 50 N push) were used in the measurement. A force plate was used to measure the push force. A 3-D scanning laser vibrometer (Polytec, 3-D PSV-500) was used to measure the distributed 3-D vibrations on the surface of the instrumented handle and on the dorsal surface of the fingers. To allow the laser beam to reach the fingers, the top part of each glove finger was cut off. As also shown in Fig. 2, the handle fixture on the 3-D vibration test system could block part of the view of the fingers coupled on the handle for a given orientation of the fixed laser vibrometer. Hence, the measurement was performed separately on the left and right hands in the two areas on the index and middle fingers, as shown in Fig. 3, assuming that the transmissibilities on the left and right hands are not significantly different under the same test conditions. To assure a good signal and to avoid the effect of hair on the measurement, a piece of retro-reflective tape was applied at each measurement point, as shown in Fig. 3. To avoid any adverse effect of the retro-reflective tape on the subject’s skin, a piece of the first-aid adhesive tape was placed between the reflective tape and the skin; this also assured a firm attachment of the reflective. Two consecutive trials of the measurement for each of the 12 test treatments (four hand forces and three glove conditions including bare hand) were performed. While the measurement on three of the subjects started on the right hand, the sequence of the treatments for each hand was randomized. In each trial, the laser vibrometer scanned the defined measurement points sequentially when the subject was comfortable performing the required actions and maintaining the required hand forces. Guided by a researcher, the fingers of each subject with and without wearing a glove were positioned on the handle at similar locations.

To obtain the glove transmissibility in each direction, the human finger transmissibility with bare hand (*T_Bare Finger_*) and with gloved hand (*T_Gloved Finger_*) were first calculated from:

(1) $$T_{\mathrm{Bare}\;\mathrm{Finger}\_i}\;(\omega )=\frac{a_{\mathrm{Bare}\;\mathrm{Finger}\_i}\;(\omega )}{a_{\mathrm{Excitation}\_i}\;(\omega )},\;\;\;T_{\mathrm{Gloved}\;\mathrm{Finger}\_i}\;(\omega )=\frac{a_{\mathrm{Gloved}\;\mathrm{Finger}\_i}\;(\omega )}{a_{\mathrm{Excitation}\_i}\;(\omega )},i=x,y,\;\mathrm{and}\;z,$$

where, *a_Excitation_* is the acceleration on the handle measured using the accelerometer fixed in the handle; *a_Bare Finger_* and *a_Gloved Finger_* are the accelerations measured on the human finger using the laser vibrometer without and with wearing a glove, respectively; and *ω* is vibration frequency in Rad/second. The calculation was performed using the program built into the data acquisition system of the laser vibrometer.

Then, the glove finger transmissibility (*T_Glove_*) in each direction was evaluated using the following formula:

(2) $$T_{\mathrm{Glove}\_i}\;(\omega )=\frac{T_{\mathrm{Gloved}\;\mathrm{Finger}\_i}\;(\omega )}{T_{\mathrm{Bare}\;\mathrm{Finger}\_i}\;(\omega )},i=x,y,\;\mathrm{and}\;z,$$

VR gloves significantly increase the effective grip size of the handle [30]. While the increased effective diameter of the handle does not change the orientation of the fingers along the handle axis in the *y*, or shear direction, it may significantly change the position and orientation of the fingers in the *x* and *z*, or compression, directions. The finger orientation may also vary with hand size and glove thickness. As a result, the glove finger transmissibility in the *x* and *z* directions evaluated using Eq. (2) may not be representative of the actual glove transmissibility in these two directions. Furthermore, it is very difficult to determine these two directions of the fingers in tool operations at workplaces, as the dimensions and positions of tool handles vary greatly among the tools. This makes it very difficult to reliably match these two finger coordinates used in the laboratory test with those used in the tool operation for estimating the direction-specific transmissibility of the glove fingers. This can be solved by considering the transmissibility of the vector sum of the vibrations in the *x* and *z* directions, because the variation of the finger orientation does not change the vector sum of the vibrations if the handle vibration does not vary significantly with the location around the handle. Because the input vibration in each direction used in the experiment is controlled over the frequency band of the test and any possible small variation of the input vibration does not change the glove transmissibility [17,21], the combined transmissibility in the *x* and *z* compression directions can be derived from the transmissibility spectra evaluated using Eq. (2) from the following formula:

(3) $$T_{\mathrm{Glove}\_xz}\;(\omega )=\frac{\sqrt{T_{\mathrm{Glove}\_x}^{2}\;(\omega )+T_{\mathrm{Glove}\_z}^{2}\;(\omega )}}{\sqrt{2}}$$

This study also considered the glove finger transmissibility of the total vibration (vector sum of the three-direction vibrations) in the estimation of the glove effectiveness like in [31]. Similar to Eq. (3), the total vibration transmissibility of glove fingers was derived from [21]:

(4) $$T_{\mathrm{Glove}\_\mathrm{xyz}}\;(\omega )=\frac{\sqrt{T_{\mathrm{Glove}\_x}^{2}\;(\omega )+T_{\mathrm{Glove}\_y}^{2}\;(\omega )+T_{\mathrm{Glove}\_z}^{2}\;(\omega )}}{\sqrt{3}}$$

### 2.2. Synthesis of the representative transmissibility spectra of glove fingers

The experimental data measured on the four points in Area 1 in Fig. 3 for each subject were averaged and used to calculate the glove transmissibility spectra for the distal fingertip area of the fingers. Similarly, those measured on the six points in Area 2 (Fig. 3) were averaged and used to calculate the glove transmissibility spectra for the proximal part of the fingers. Then, the transmissibility spectra of these two parts were further averaged and used to represent the overall transmissibility spectra of the full fingers for each subject. Finally, the spectra of all the subjects were averaged and used to represent the general spectrum for the estimation of the tool-specific effectiveness of the glove fingers.

While the transmissibility spectra measured with the 15 N grip were significantly different from those measured with the other three hand forces (*p <* 0.001), the remaining spectra were similar [21]. Therefore, the spectra measured with the 30 N grip and 50 N push were selected for the estimation of the general effectiveness of the glove fingers, which is consistent with the practice used in the standard test and evaluation of AV gloves [12].

Because of the structural limitations of the 3-D vibration test system [32], only the spectra from 16 to 500 Hz were measured in the reported experiment [21] and in the current study. In principle, the glove transmissibility at lower frequencies is very close to unity [24]. This has also been confirmed from the experimental data [18,21,28]. Hence, the missed spectra from 6.3 to 16 Hz were assumed to be a linear interpolation between the unity transmissibility value (1.0) at 6.3 Hz and that measured at 16 Hz. The components over 500 Hz are unlikely to affect the transmissibility values of the frequency-weighted vibration required for the risk assessment of the vibration exposure in the current standards [3,4], as the frequency weighting over 500 Hz is below 0.0314 and the major vibration components of tools are usually below 500 Hz [33]. Because of the lack of data from 500 to 1,250 Hz, the unweighted acceleration was calculated from 6.3 to 500 Hz in this study.

### 2.3. Tool vibration spectra

**Fig. 4:**
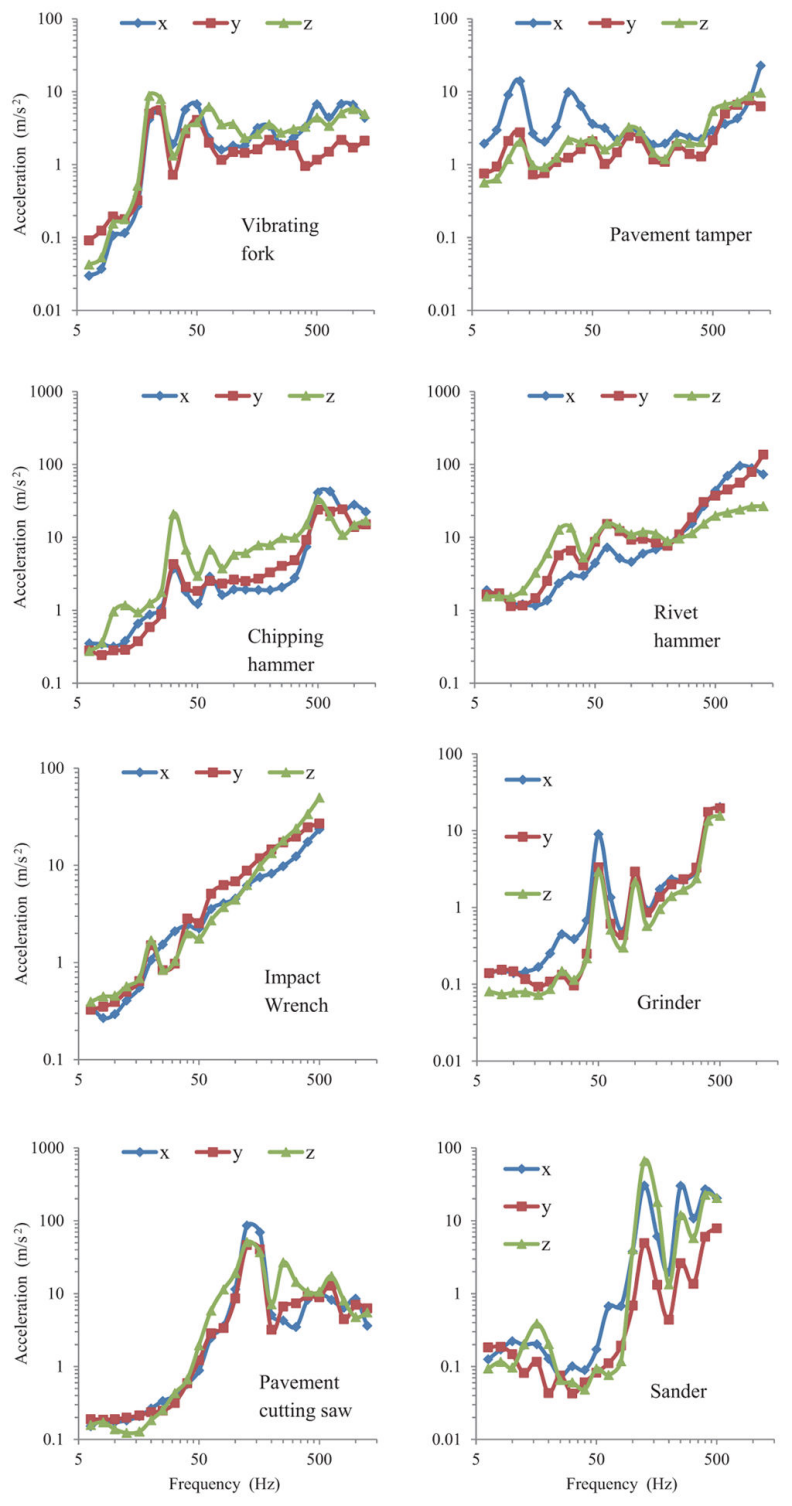
Examples of tool vibration spectra in three directions (*a_x_, a_y_, a_z_*) used in this study.

Over seventy spectra of tool vibrations used in a previous study of the gloved palm response to tool vibration [28] were analyzed relative to the finger response for the current study. While many of the tool spectra compiled for [28] came from studies that measured several tools in controlled trials, several of the included spectra were collected in the field or lab with few replicate trials. Several other tools from the literature including a vertical grinder [34], a hand-held grinded workpiece [35], and rivet bucking bars [36], with more vibration distributed in shear were also analyzed. Overall, the tool spectra were selected based on the following criteria: the vibration spectra were measured using the standard method defined in ISO 5349-1 (2001) [3], such that the spectra were simultaneously measured in the three orthogonal translational directions; the directions were documented such that the *x*, *y*, and *z* axes could be matched with the directions used to collect the glove transmissibility spectra – thus allowing the shear and compression directions to be confirmed; the measurements from impact tools were only usable if there was no significant dc shift [1]. The spectra were expressed in the one-third octave bands from 6.3 to 1,250 Hz, although only the data from 6.3 to 500 Hz were used in this study. If the original spectra did not include the full frequency range of concern, the missing values were taken as zero. As the reported tool vibration spectra are the mean spectra, they were directly used in this study with no further statistical analysis. Some examples of the tool vibration spectra are shown in Fig. 4.

### 2.4. Calculation of tool-specific glove transmissibility values

Similar to the method used in ISO 10819 [11,12], the transmissibility values of frequency-weighted accelerations in the combined *x* and *z* compression directions (*T_w-xz_*) and in the *y* shear direction (*T_w-y_*) were calculated from

(5) $$\begin{array}{l} T_{w-xz}=\frac{\sqrt{\sum_{i}T_{xz}^{2}\;(\omega_{i})\cdot [a_{x}^{2}\;(\omega_{i})+a_{z}^{2}(\omega_{i})]\cdot W_{h}^{2}\;(\omega_{i})}}{\sqrt{\sum_{i}\left[a_{x}^{2}\;(\omega_{i})+a_{z}^{2}(\omega_{i})]\cdot W_{h}^{2}\;(\omega_{i}\right)}},\\ T_{w-y}=\frac{\sqrt{\sum_{i}{\left[T_{y}\;(\omega_{i})\cdot a_{y}\;(\omega_{i})\cdot W_{h}\;(\omega_{i})\right]}^{2}}}{\sqrt{\sum_{i}{\left[a_{y}\;(\omega_{i})\cdot W_{h}\;(\omega_{i})\right]}^{2}}}, \end{array}$$

where *T_xz_* and *T_y_* are the glove vibration spectra, *a_x_*, *a_y_*, and *a_z_* are tool vibration spectra in three orthogonal directions, *W_h_* is the frequency weighting factor for hand-arm vibration exposure defined in ISO 5349-1 (2001) [3], and *ω_i_* is the vibration frequency in Rad/s corresponding to 6.3 to 500 Hz in the one-third octave bands.

Similarly, according to the total vibration (vector sum of the three-axial vibrations) defined in ISO 5349-1 (2001) [3], the transmissibility value for total vibration was calculated from [31]:

(6) $$T_{w-\mathrm{xzy}}=\frac{\sqrt{\sum_{i}\left\{T_{xz}^{2}\;(\omega_{i})\cdot [a_{x}^{2}\;(\omega_{i})+a_{z}^{2}\;(\omega_{i})]+T_{y}^{2}\;(\omega_{i})\cdot a_{y}^{2}\;(\omega_{i})\}\cdot W_{h}^{2}\;(\omega_{i}\right)}}{\sqrt{\sum_{i}\left\{a_{x}^{2}(\omega_{i})+a_{y}^{2}(\omega_{i})+a_{z}^{2}(\omega_{i})]\cdot W_{h}^{2}(\omega_{i}\right)}}$$

The unweighted transmissibility values (*T_u_*) were also calculated using Eqs (5) and (6) by taking the weighting (*W_h_*) as unity (1.0) for each frequency. After the total transmissibility value was obtained, the percent reductions for weighted vibration (*R_w_*) and unweighted vibration (*R_u_*) were respectively calculated for all of the tools using the following equations:

(7) $$R_{w}=(1-T_{w})\cdot 100\%,\;\;\;R_{u}=(1-T_{u})\cdot 100\%$$

## 3. Results

### 3.1. Vibration transmissibility spectra of the VR gloves

**Fig. 5:**
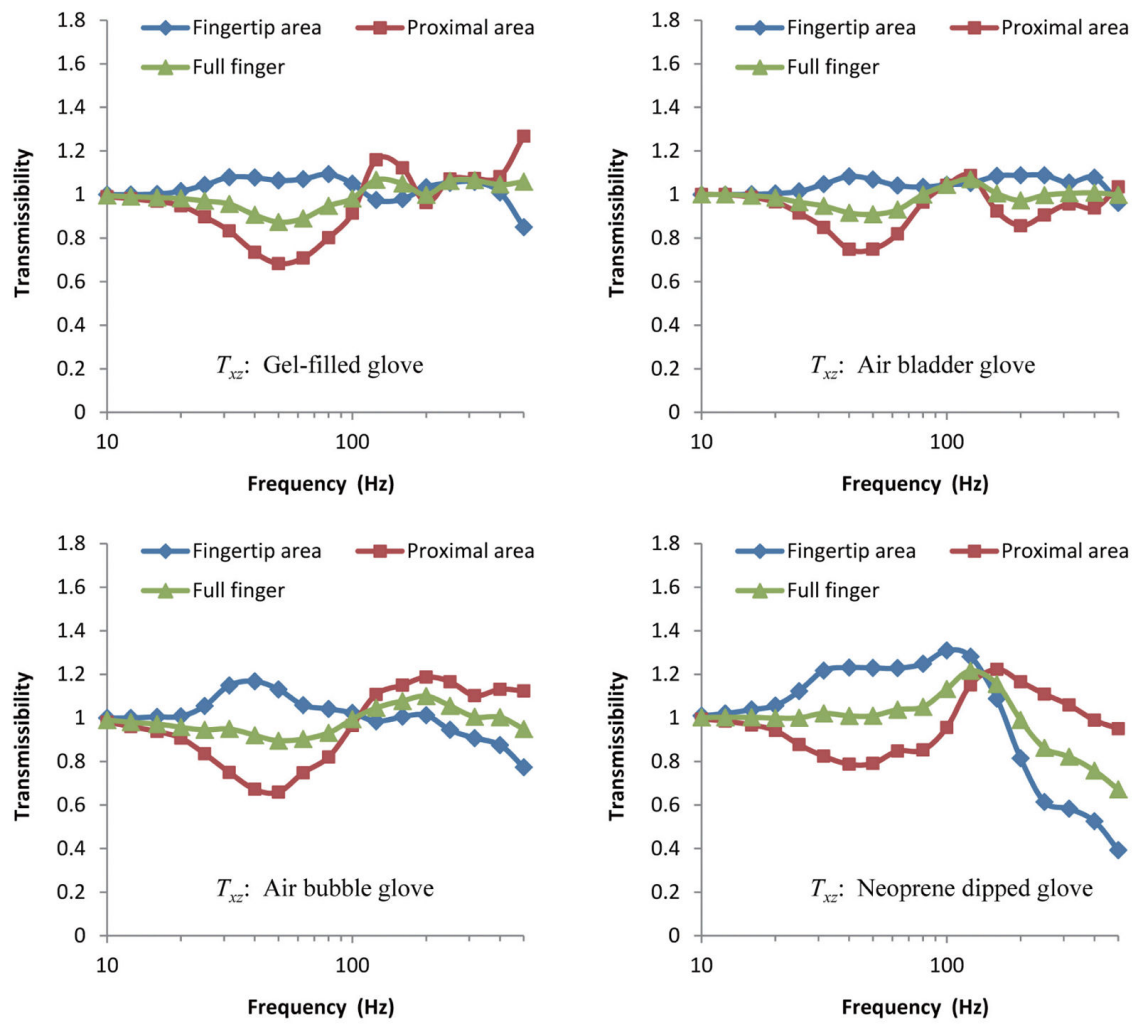
Vibration transmissibility spectra of the glove fingers in the combined *x* and *z* compression directions (*T_xz_*).

Figure 5 shows the glove transmissibility spectra of the vibrations in the combined x and z directions, which are synthesized primarily based on the data calculated using Eq. (3). The spectrum for the fingertip area is the average of the spectra measured on the four points in Area 1 shown in Fig. 3. Similarly, the spectrum for the proximal area is the average of the spectra measured on the six points in Area 2. The spectrum for the full finger is the further average of the spectra for the two areas.

At less than 25 Hz, the transmissibility for the gloves is close to unity (1.0). While the gel and air bladder gloves slightly amplify the input at the fingertips through to 400 Hz, the bubble glove peaks at a higher magnitude at around 40 Hz and gradually declines before attenuating the vibration at frequencies above 250 Hz. The neoprene glove responds more sharply with a resonance at 100 Hz; it attenuates the vibration at frequencies higher than its peak. The general trends are different in the proximal area of the fingers. The gloves all tend to reduce the vibration in the proximal area up to about 100 Hz, but amplify the input at higher frequencies. The gel, bubble and neoprene gloves amplified the vibration throughout most of the range from 100 to 500 Hz, while the air bladder glove reduces the vibration at frequencies higher than its 125 Hz peak.

**Fig. 6:**
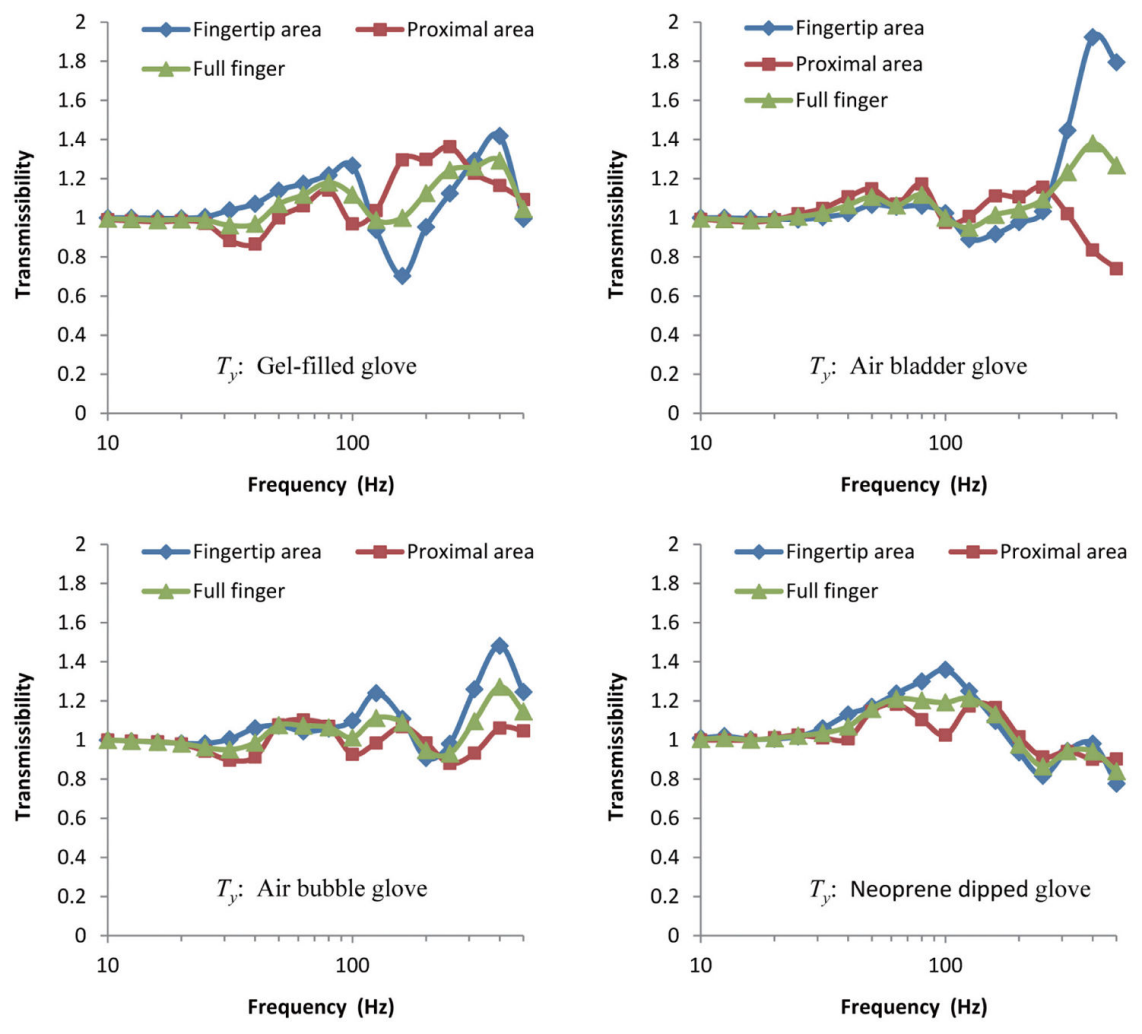
Vibration transmissibility spectra of the glove fingers in the *y* shear direction (*T_y_*).

Figure 6 shows the transmissibility spectra of the glove fingers in the *y*, or shear, direction, which are calculated using Eq. (2). The air bladder glove has a substantial resonance at 400 Hz at the fingertips at nearly twice the input. The gel and bubble gloves each had two distinct peaks, first at 100 and 125 Hz, respectively, and also at 400 Hz. The neoprene glove peaked at 100 Hz, gradually decreasing in magnitude to below unity at 200 Hz. In the proximal area, the gel glove amplified the vibration throughout much of the range tested above 50 Hz. The air bladder, air bubble and neoprene gloves tended to be closer to unity from 50 to 500 Hz with the bladder and neoprene gloves attenuating the input over 200 Hz.

**Fig. 7:**
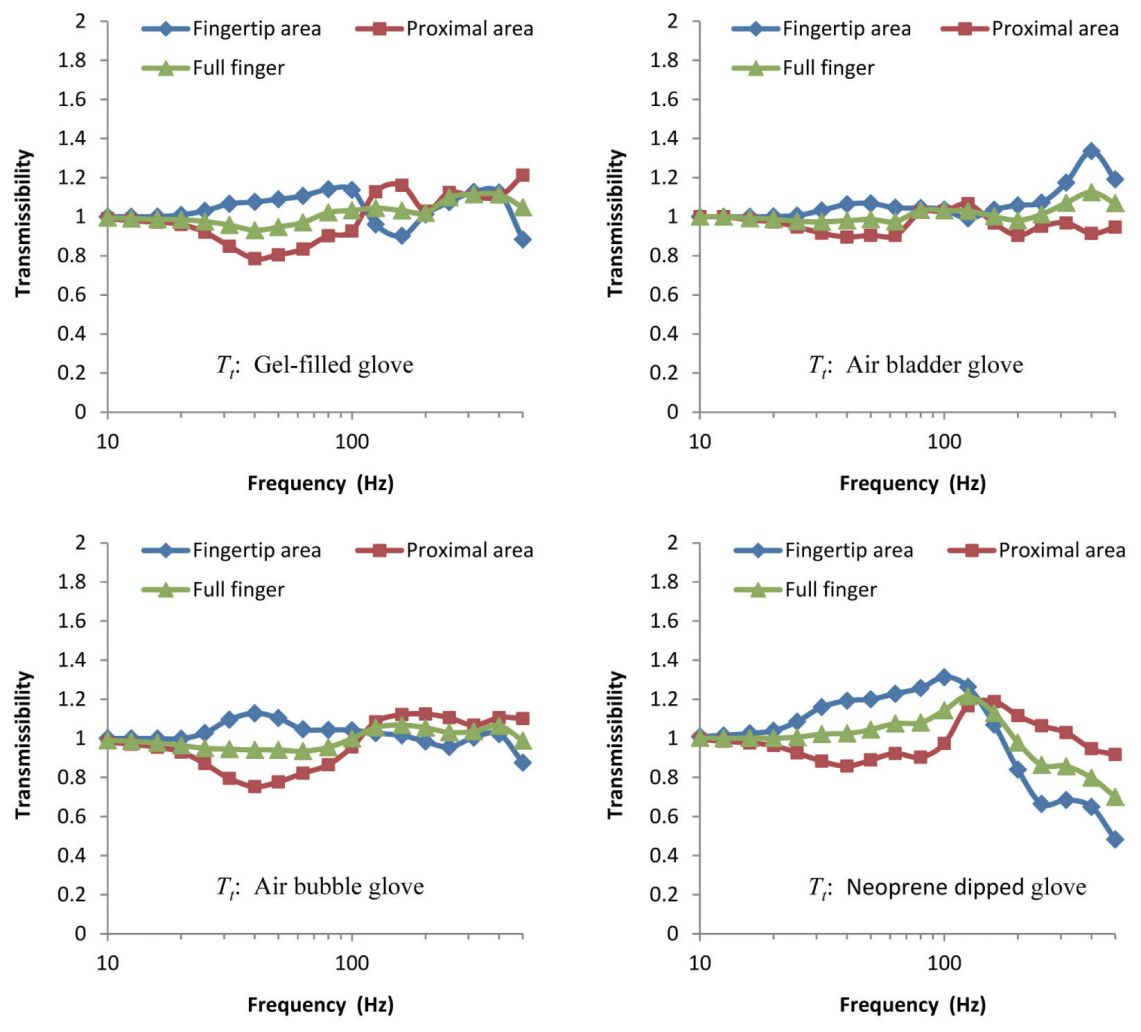
Vibration transmissibility spectra of the glove fingers for total vibration (vector sum of the accelerations in *x, y*, and *z* directions) (*T_xyz_*).

Figure 7 shows the glove transmissibility spectra of the total vibration, which are the results calculated using Eq. (4). Their trends are similar to those shown in Fig. 5. The vibrations in the combined directions take about two thirds of the weight in the vector summation of the three axial vibrations, although the response to shear for the fingertips does dominate at high frequencies for the air bladder glove and slightly so for the bubble and gel glove.

### 3.2. Tool-specific transmissibility values of the VR gloves

Table 3 summarizes the average unweighted and weighted transmissibilities and accelerations at the fingertips and proximal locations for each of the gloves calculated using Eqs (5) and (6) for the examples of tool spectra shown in Fig. 4. Two representative low frequency tools – a vibrating manure fork and a paving tamper – were chosen due to their resonances being below 25 Hz. Three impact tools – a chipper hammer, rivet hammer and impact wrench – and three higher frequency tools – a pavement cutting saw, a disc angle grinder and a palm sander – were also analyzed. The weighted transmissibilities for most of the glove and tool combinations at the fingertips were above unity, with only a few slight reductions of up to 3% as presented in Table 3. The gloves amplified all of the unweighted vibration at the fingertips for the low frequency tools (vibrating manure fork, paving tamper), but tended to dampen the vibration in the x and z directions in the proximal area.

The gloves had varying levels of effectiveness for the impact and higher frequency tools. The neoprene glove was able to attenuate both the shear (Ty) and compression (Txz) vibration for the impact tools and grinder in terms of the unweighted vibration. However, due to its resonance around 100 to 125 Hz the neoprene glove was less effective for the sander and pavement cutting saw. The neoprene glove tended to amplify most of the tool vibrations in terms of the weighted transmissibility, particularly at the fingertips. The air bladder glove amplified the shear vibration at the finger tips as much as 75%, while the bubble glove increased it as much as 30% and the gel glove up to 21% for the impact and higher frequency tools. The air bubble glove was able to reduce the unweighted vibration in compression at the fingertips, but increased it in the proximal area. The air bladder glove was the opposite in that it marginally (about 5%) increased the unweighted vibration in compression (Txz) at the tips and slightly decreased it for the proximal locations. The gel glove marginally reduced the compression vibration at the fingertips, but amplified the shear and compression in the proximal area due to the peaks around 100 Hz that coincided with several tools’ operating frequencies pictured in Fig. 4.

### 3.3. Full finger reductions for all of the sampled tool spectra

Table 4 contains the total vector summed percent reductions in transmissibilities averaged over all of the fingertip and proximal locations of the fingers for all of the tool and glove combinations. Positive values in Table 4 represent attenuations of the tool vibration by the gloves and negative values are amplifications of the tool vibration by the gloves. Of the 79 tool spectra processed, 27 of the tools had their total averaged finger transmissibility reduced by more than 10% by the neoprene glove in terms of unweighted vibration. The neoprene glove reduced only three tools by more than 10% in terms of the frequency-weighted calculation, and, in fact, could amplify 10 of the tools by more than 10%. Only the chainsaw and scabbler vibrations were reduced more than 5% by any of the air bladder, air bubble, and gel gloves in terms of unweighted vibration, while the rock drill, hand-held grinded golf club head, scabbler and chainsaw could be reduced in terms of the frequency-weighted vibration for any of those three gloves. The gloves marginally reduced (*<* 5%) the total finger vibration for the low frequency tools primarily due to reductions in the proximal area. Some tools, including the needle scaler, chisel of the stone hammer, vertical grinder from the shipyard, and some of the rivet bucking bars increased the unweighted vibration with the gel and air bladder gloves more than 10%. The air bubble glove was close to unity or amplified the tool vibration at that level for several of the same tools. The needle scaler, stone hammer chisel, vertical grinder, and sampled bucking bars all have proportionately larger acceleration magnitudes in shear as shown in Table 5.

## 4. Discussion

It is relatively difficult to protect the fingers from vibration with gloves compared to the palm. The fingers have a much lower individual effective mass and have much less cushioning and damping than the palm, and naturally are much stiffer [24]. It is therefore much more challenging to create or augment – as in the case of the palm – an impedance mismatch with a tool handle sufficient to reduce the vibration. This study examined the tool-specific effectiveness of vibration reducing gloves at the fingers. The results can be used to help select appropriate gloves for use with vibrating tools.

### 4.1. Mechanisms of the air bubble and neoprene gloves in both areas of the fingers

This study gave further insight into the mechanisms of how the fingers and gloves interact. As discussed at length in [21] for the air bladder and gel gloves, the glove response depends on both the relatively uniform contact pressure distribution and contact stiffness at the fingertips and the stiffness of the finger structures (i.e. the knuckles and phalanges) in combination with the glove proximally. For instance, the bulkier gloves that require more grip effort – the gel and bubble gloves – have higher transmissibilities in the proximal area. They are more likely to bunch up at the knuckles creating areas of higher localized stiffness. The neoprene glove conforms to the circular handle due to designed ribbing and the bunching is limited. However, the stiffer, less damped material yields a sharper resonance than the other gloves. The transmissibilities for the example tools presented in Table 3 were generally higher for the neoprene glove in compression in the proximal area than at the fingertips where it was fairly effective for most tools. The neoprene glove was poor for tools with dominant operating frequencies near its 100 Hz resonance such as the pavement cutting saw and sander. The air bubble glove was able to reduce the unweighted vibration in compression at the fingertips for the impact and higher frequency tools. However, like the air bladder glove, the bubble glove increased the shear vibration at the fingertips; though, generally half as much. The increase in shear may be a combination of the thicker glove with the lack of effective mass and stiffness and damping in the shear direction. The bubbles may add a minor structural element in shear that is lacking for the bladder glove.

### 4.2. Tool and glove matches

The neoprene glove has the potential to cut vibration from 27 of the 79 tools more than 10% when considered in terms of the unweighted vibration calculation as shown in Table 4. The greater than 10% levels of reduction or amplification chosen to delineate the effectiveness of the tool and glove combinations are subjective rather than statistically defined. However, half of the tools cited in our previous study based on the tool specific frequency response at the palm reduced vibration more 10% with at least one of the gloves and half reduced it less than 10% [28]. The relationship of transmissibility to the health effects requires further research. The neoprene glove was most effective for impact and higher frequency tools that didn’t operate primarily near the glove’s resonance. However, because of the significant amplification at frequencies below its peak in compression, the performance of the neoprene glove rates poorly when applying the frequency weighting for the assessment.

The air bladder, bubble, and gel gloves reduced the input from only a couple of tools in terms of the unweighted or weighted vibration, and amplified several tools’ vibration more than 10%. All three gloves perform poorly in the shear direction and they all tend to amplify the vibration in the compression directions at just over unity for much of the frequency band tested, depending on the finger location. The total finger vector sum is weighted to the compression but the level of acceleration in the shear direction for many of the tools with higher frequency inputs can be high.

Most of the tools tested don’t exhibit dominant axial shear vibration along the hand held portion of the tool, however there are a few exceptions where it can be a factor: the needle scaler; chisel of stone hammer; angle grinder; rivet bucking bars; hand-held workpiece. The directional and regional transmissibilities and acceleration magnitudes for some of those tools are tabulated in Tables 3 and 5. The chisel and handle of the stone hammer were very good examples of the influence of axial shear. The sliding action of the chisel along its axis while gripped with the hand has different frequency content at higher frequencies at a high magnitude compared to that of the tool’s handle [23,28,37]. The neoprene glove was relatively effective for holding the chisel compared to the other gloves which amplified the vibration more than 10%; however, at the handle, the gloves increased the vibration at the fingertips but decreased it in the proximal area and, thus, the average for each was close to the bare hand.

Many models of needle scalers are held along the tool’s body, which holds the needles. Though some are available with a pistol grip, the main body still requires a firm grip. Therefore, its primary action is in axial shear. The model tested had a dominant frequency of 80 Hz in the y direction [37]. All of the gloves increased the vibration from the needle scaler and cannot be recommended. The tool requires some other intervention.

The action of the vertical grinder measured at the shipyard, like other higher frequency tools, is distributed in all three orthogonal directions. Because of its high magnitude in all three directions the axial shear vibration was powerful. The neoprene glove was the only one effective at reducing its vibration. The air and gel gloves match the peaks of its shear vibration poorly and likely cannot help protect the fingers. These observations apply to the handheld grinding of workpieces such as golf club heads.

The steel and tungsten rivet bucking bars in Tables 4 and 5 were assessed in the field tests on airplane frames in our reported study [36]. Rivet hammer H in Table 4 was used to set the rivets with the bars. The primary action of the bars was in shear, sliding along or across the fingertips and proximal area of the fingers; sometimes the workers also limited the sliding by using their palms. As shown in Table 5, the transmissibilities were very similar between the two bars; the primary difference was the tungsten bar transmitted less than half the magnitude that the steel bar did. Again, like many of the aforementioned tools, the recommendation depends on the whether the unweighted or weighted calculations are used. The technique for holding the bar against the rivet will also make a difference in which glove could be chosen – whether it’s held at the fingertips, using the entire finger, or the palm. In terms of the total finger vibration the air and gel gloves would not be recommended if the unweighted vibration is used for the evaluation because the gloves amplify the dominant shear vibration. In terms of the weighted vibration, the air and gel gloves are roughly equivalent to the bare hand, while the neoprene glove increases the vibration. In general, the gloves tested are not an improvement over the bare hand when used with rivet bucking bars. Their effectiveness will depend on the technique used to hold the bar – whether at the fingertips, over the entire fingers, or pressed by the palm.

### 4.3. Comparison to palm

The neoprene glove was the most effective at the fingers as indicated in the Tables 3, 4, and 5. While the air bladder glove was the most effective in terms of the standardized glove test [17], the gloves, on average, reduced the tool vibration to similar levels when assessed in the three orthogonal directions simultaneously [28]. The bubble and bladder gloves were comparable in 3D. The neoprene glove was near the median for effectiveness at the palm. Although it did not have as high a response in the shear directions as the air gloves, it also responded around 500 Hz in shear and was not as effective in the z direction along the axis of the forearm.

### 4.4. Unweighted vs. weighted acceleration

The standardized glove assessment [12] at the palm uses the frequency weighted vibration to screen the gloves. While the weighted acceleration may be suitable for palm exposures, it may not be appropriate for the fingers which respond at higher frequencies [38]. Assessing the vibration based on unweighted or weighted vibration in this study can lead to different conclusions as shown in Table 4. In terms of unweighted vibration, the neoprene glove reduced the total vibration into the fingers more than 10% for 27 of the 79 tools, with 2 tools amplified more than 10%. In terms of weighted vibration, the neoprene glove can only reduce the vibration from three tools more than 10% and it amplifies 9 tools’ inputs more than 10%. Several of the tool interactions with the air bladder and gel gloves are also different between the unweighted and weighted assessment. Although the trends are often the same from either analysis for either glove, the scale of the amplifications is generally lower with the weighted assessment. The weighted vibration can dull the influence of the high frequency peak of the air glove in the shear direction in particular. As mentioned in the Methods, the weighting can have a strong influence on frequencies in the frequency range of the peak of the air gloves’ responses in shear, with the weighting factor at less than 0.0314 for 500 Hz [3]. The weighted assessment is perhaps too conservative at the fingertips but can give insight into the tool exposure in terms of the frequency content of the tool when used in concert with the unweighted frequency response.

### 4.5. Field measurement considerations

It is very important to document the dominant direction of vibration for the tool relative to the hand in lab and field measurements of tool vibration [3]. Several of the tools considered have significant vibration in all three directions or act primarily in the shear direction. Some of the gloves are ineffective against vibration in shear. Therefore, it is important to document that axial shear direction relative to the direction of the dominant action of the tool when setting the accelerometer near the hand contact with the tool. This consistency for the choice of the axial shear direction is especially important for tools such as bucking bars, chisels, and scalers. When the vibration is distributed in all three directions, as in the case of the vertical grinder, the axial shear direction along the axis of the hand/handle interface would be the dominant direction.

### 4.6. Limitations

There are several limitations to this investigation. The most important limitation is that the glove finger transmissibility was limited to the frequency range of 16 to 500 Hz but the required frequency range for assessing hand-transmitted vibration is from 6.3 to 1,250 Hz in the one-third octave bands (ISO 5349-1, 2001). The lack of the glove transmissibility spectra for frequencies less than 16 Hz is not a significant issue, as the vibration transmissibility of the gloves at such low frequencies is very close to unity. The lack of the spectra at more than 500 Hz is not important either when the frequency-weighted acceleration is used to assess the effectiveness of the gloves, as there is less than 1% weighting at more than 500 Hz in the current standard frequency weighting function [3]. However, some studies suggest that the current frequency weighting greatly underestimates the effect of high frequency vibration on the development of vibration-induced finger disorders [39–43]. A few other studies suggested that it could be better to use a unit weighting (unweighted acceleration) or other alternative finger frequency weightings [37,38,44,45]. If such alternative weightings are used for assessing the glove effectiveness, the vibration components at frequencies above 500 Hz cannot be ignored in many cases. For example, significant vibration components can be observed in the range of 500 to 1,250 Hz on the percussive tools, bucking bars, and golf club heads [35,36]. If the unit weighting is used, the gloves will appear to be more effective than their percent reductions shown in Table 4. However, such an underestimation is unlikely to change the rank orders of the gloves’ effectiveness considered in this study. Hence, the listed percent reduction should still have value for the selection of the gloves for protecting the fingers.

Another limitation is that only four types of gloves were tested. From previous testing [17,18] there was little variation in spectra between gloves that used the same mechanisms. The air bladder gloves had similar responses; the gel gloves were also similar; the air bubble gloves were comparable to one another. Also, the number of samples of spectra compiled for the various tools varied from 1 or 2 sample spectra, to studies with up to one hundred examples. Therefore no statistics were done for the transmissibilities. Different participants were used to measure the two sets of gloves between this and the previous study [21]. However, two of the participants were involved in both studies and the anthropometric measurements for both groups of subjects were similar as shown in Table 2.

### 4.7. Recommendations

Given the advantages of the air bladder or bubble glove at the palm and the neoprene glove at the fingers for several tools, a possible improvement would be to make a glove with the combination of an air bladder or bubbles at the palm and neoprene fingers. The neoprene glove could use a material with a higher level of damping to lessen its resonance peak. However, such a change could have adverse effects such as broadening the frequency range at which the glove amplifies the input and would require tool-specific vibration testing to evaluate its potential effectiveness. Such a material may also degrade more quickly than the dipped neoprene.

## 5. Conclusions

This study investigated the effectiveness of VR gloves for reducing the vibration transmitted to the fingers. The results confirmed that the effectiveness varied with the gloves and the vibration reduction of each glove depended on tool, direction, and finger location. VR gloves, including certified anti-vibration gloves do not provide much vibration reduction when judged based on frequency-weighted acceleration. However, some of the VR gloves can provide more than 10% reduction of the unweighted vibration on some tools or workpieces. Tools and gloves can be matched for better effectiveness for protecting the fingers.

## Figures and Tables

**Table 1:** Glove specifications

Glove	Glove mechanism	Standard assessment (ISO 10819, 2013 [12])	Grip strength reduction
A	Viscoelastic Gel	Not classified as AV glove	40%
B	Air Bladder	Classified as AV glove	30%
C	Air Bubble	Marginally classified as AV glove	34%
D	Neoprene-dipped	Not classified as AV glove	26%

**Table 2:** Subject Anthropometry for tests of air bubble and neoprene-dipped gloves in the current study. The averaged anthropometries for the subjects which were tested using the air bladder and viscoelastic gel gloves in [21] are tabulated too. The Hand Sizes of all Subjects (calculated by EN 420 [29]) in the current study are 9 and the mean Hand Size in [21] was 9

Subject	Hand length (mm)	Hand circumference (mm)	Stature (cm)	Weight (kg)
1	193	231	183	95.7
2	199	215	191	78.1
3	198	208	182	84.6
4	203	217	185	71.6
5	199	215	180	82.2
6	198	219	180	121.8
Means	198.3	217.5	183.5	89
st dev	3.20	7.58	4.14	17.94
Welcome et al. [21]	193.7	214	180.9	88.4

**Table 3:** Examples of the glove vibration transmissibilities on tools in the fingertips and proximal areas of the fingers. A*_y_* and T*_y_* are the acceleration magnitude and transmissibility in shear; A*_xz_* and T*_xz_* are the acceleration magnitude and transmissibility in compression; A*_t_* and T*_xzy_* are total vector sums of the acceleration magnitudes and transmissibilities. Tool accelerations in m/s^2^; Transmissibilities unit less

	Unweighted			Weighted			Unweighted			Weighted		
	Ay	Axz	At	Ay	Axz	At	Ay	Axz	At	Ay	Axz	At
	Vibrating fork						Pavement tamper					
(m/s^2^)	10.35	23.02	25.24	5.68	10.74	12.15	14.25	29.68	33.20	10.17	12.67	18.19
Fingertip	Ty	Txz	Txzy	Ty	Txz	Txzy	Ty	Txz	Txzy	Ty	Txz	Txzy
Gel	1.05	1.02	1.03	1.01	1.03	1.03	1.06	1.02	1.03	1.01	1.01	1.01
Air Bladder	1.05	1.03	1.04	1.00	1.02	1.01	1.10	1.04	1.05	1.00	1.01	1.01
Air Bubble	1.03	1.02	1.02	0.99	1.04	1.03	1.06	1.01	1.02	1.00	1.02	1.01
Neoprene	1.06	1.04	1.05	1.03	1.11	1.09	1.07	1.06	1.06	1.03	1.07	1.04
Proximal												
Gel	1.03	0.94	0.96	0.98	0.90	0.92	1.02	1.00	1.00	0.98	0.97	0.97
Air Bladder	1.05	0.91	0.94	1.02	0.92	0.94	1.01	0.97	0.97	1.00	0.99	0.99
Air Bubble	0.99	0.92	0.93	0.97	0.85	0.88	0.98	1.00	0.99	0.99	0.96	0.96
Neoprene	1.05	0.93	0.95	1.03	0.90	0.93	1.02	1.01	1.01	1.01	0.98	0.99
	Chipping hammer							Rivet hammer				
(m/s^2^)	27.68	67.86	73.30	2.70	11.84	12.16	92.25	143.34	170.46	7.71	18.92	20.44
Fingertip	Ty	Txz	Txzy	Ty	Txz	Txzy	Ty	Txz	Txzy	Ty	Txz	Txzy
Gel	1.08	0.94	0.96	1.07	1.07	1.07	1.13	0.98	1.02	1.08	1.04	1.05
Air Bladder	1.71	1.00	1.13	1.11	1.05	1.05	1.62	1.03	1.24	1.15	1.03	1.05
Air Bubble	1.25	0.89	0.95	1.06	1.12	1.11	1.25	0.90	1.01	1.09	1.04	1.05
Neoprene	0.86	0.71	0.74	1.07	1.19	1.18	0.91	0.73	0.79	1.09	1.12	1.12
Proximal												
Gel	1.12	1.15	1.15	0.98	0.86	0.87	1.16	1.12	1.13	1.08	0.91	0.94
Air Bladder	0.82	0.98	0.96	1.03	0.87	0.88	0.89	0.97	0.95	1.03	0.92	0.94
Air Bubble	1.03	1.06	1.06	0.96	0.80	0.81	1.02	1.10	1.08	1.00	0.89	0.91
Neoprene	0.92	0.95	0.95	1.02	0.86	0.87	0.94	1.01	0.99	1.05	0.93	0.94
	Impact wrench							Vertical grinder				
(m/s^2^)	36.53	86.26	93.68	3.23	5.73	6.58	154.84	136.14	206.18	6.30	9.41	11.33
Fingertip	Ty	Txz	Txzy	Ty	Txz	Txzy	Ty	Txz	Txzy	Ty	Txz	Txzy
Gel	1.15	0.96	0.99	1.08	1.02	1.04	1.16	0.96	1.08	1.20	1.03	1.08
Air Bladder	1.63	1.03	1.14	1.14	1.05	1.07	1.75	1.03	1.48	1.61	1.05	1.25
Air Bubble	1.26	0.86	0.93	1.09	0.99	1.01	1.29	0.87	1.13	1.25	0.98	1.07
Neoprene	0.92	0.59	0.65	1.09	0.98	1.01	0.86	0.66	0.78	0.91	1.14	1.08
Proximal												
Gel	1.16	1.15	1.16	1.06	1.00	1.01	1.15	1.13	1.14	1.19	0.98	1.05
Air Bladder	0.90	0.98	0.97	1.04	0.94	0.96	0.84	0.99	0.91	0.92	1.02	0.99
Air Bubble	1.02	1.12	1.11	0.99	1.01	1.01	1.03	1.11	1.06	1.00	1.01	1.01
Neoprene	0.95	1.01	1.00	1.04	1.00	1.01	0.91	0.98	0.94	0.92	0.97	0.95
	Pavement cutting saw						Sander					
(m/s^2^)	46.10	94.97	105.58	5.30	10.90	12.12	11.59	94.63	95.34	0.81	9.87	9.90
Fingertip	Ty	Txz	Txzy	Ty	Txz	Txzy	Ty	Txz	Txzy	Ty	Txz	Txzy
Gel	0.98	0.99	0.99	0.99	0.99	0.99	1.13	0.98	0.98	1.02	0.98	0.98
Air Bladder	1.08	1.06	1.07	0.97	1.06	1.04	1.66	1.05	1.06	1.15	1.05	1.06
Air Bubble	1.18	0.98	1.02	1.16	1.00	1.03	1.30	0.95	0.95	1.22	0.98	0.98
Neoprene	1.12	1.11	1.11	1.20	1.20	1.20	0.95	1.06	1.06	1.14	1.23	1.23
Proximal												
Gel	1.17	1.12	1.13	1.11	1.10	1.10	1.12	1.15	1.15	1.07	1.15	1.15
Air Bladder	1.04	1.00	1.01	1.05	1.02	1.02	0.86	1.03	1.03	0.98	1.07	1.07
Air Bubble	1.00	1.12	1.10	1.01	1.09	1.07	1.03	1.12	1.12	1.00	1.11	1.11
Neoprene	1.10	1.15	1.14	1.14	1.13	1.13	0.96	1.11	1.11	1.09	1.15	1.15

**Table 4:** Percent reduction of the total vibration on tools (negative value: the glove amplifies the vibration transmitted to fingers; positive value: the glove reduces the vibration transmitted to fingers). G-A – Gel Glove; G-B – Air Bladder Glove; G-C – Air Bubble Glove; G-D – Neoprene Glove. All tools are cited from [28,34–36]

Tool or process	Aw m/s2	Unweighted %				Mean	Weighted %				Mean
		G-A	G-B	G-C	G-D		G-A	G-B	G-C	G-D	
Fast Fork-Tines	26.56	1	0	3	3	2	3	0	4	0	*2*
Fast Fork – Wire Mesh	12.15	1	2	3	2	*2*	3	2	5	−1	*2*
Slow Fork-Tines	7.99	−1	0	1	5	*1*	2	2	4	0	*2*
Slow Fork – Wire Mesh	3.92	−1	0	2	5	*2*	3	2	4	−1	2
Hand-held Tractor – Rota filling	6.80	4	4	5	−3	*3*	4	4	5	−2	3
Hand-held Tractor – Rota pudding	5.27	3	3	4	−4	*2*	4	4	5	−2	*3*
Hand-held Tractor – Transportation	8.15	2	2	3	−2	*1*	2	2	3	−1	*2*
Bench Rammer	30.50	1	0	2	−5	−*1*	2	0	2	−1	*1*
Floor Rammer	23.69	−2	0	−1	0	−*1*	1	1	0	−1	*0*
Pavement Tamper	18.19	−1	−1	0	−2	−*1*	1	0	1	−1	0
Impact Wrench A	6.58	−7	−4	−2	19	*2*	−2	−1	0	0	−*1*
Impact Wrench B	2.69	−8	−7	−4	5	−*4*	1	1	2	−2	*1*
Impact Wrench C	9.01	−9	−10	−5	8	−*4*	−1	−1	1	−5	−2
Impact Wrench D	8.09	−10	−11	−6	6	−*5*	−3	−3	−1	−8	−*4*
Rivet A	20.44	−7	−8	−4	13	−*2*	1	1	3	−2	*1*
Rivet B	15.80	−8	−4	−2	0	−*4*	−3	−2	−1	−8	−*4*
Rivet C	27.54	−7	−10	−6	10	−*3*	1	−1	1	−5	−*1*
Rivet D	29.11	−8	−11	−6	9	−*4*	2	1	3	−3	*1*
Rivet E	13.48	−6	−3	−1	12	*0*	−1	0	1	−6	−*2*
Rivet F	17.58	−8	−10	−5	11	−*3*	2	1	3	−3	*1*
Rivet G	20.55	−5	−2	−2	9	*0*	1	0	2	−5	−*1*
Rivet H	20.65	−5	−3	−1	11	*0*	2	0	2	−4	*0*
Rivet S1	2.81	−6	−5	−4	3	−*3*	−2	−2	−2	−7	−*3*
Rivet S2	2.37	−6	−5	−3	8	−*2*	0	−1	1	−5	−*1*
Rivet S3	2.52	−6	−6	−2	11	−*1*	0	−1	1	−5	−*1*
Rivet S4	5.22	−8	−8	−4	11	−*2*	1	1	2	−3	*0*
Steel Bucking Bar B	14.86	−12	−16	−9	2	−*9*	−1	−3	0	−6	−*2*
Tungsten Bucking Bar E	6.51	−12	−14	−7	0	−*8*	0	−1	1	−5	−*1*
CH Bucking Bar	15.39	−9	−14	−7	8	−*6*	1	1	3	−3	*1*
DF Bucking Bar	27.74	−7	−6	−4	3	−*4*	1	1	3	−4	*0*
Foot Bucking Bar	12.50	−15	−18	−10	1	−*11*	−3	−5	−2	−9	−*5*
L Bucking Bar	7.65	−11	−11	−7	6	−*6*	−7	−4	−5	−9	−*6*
TR Bucking Bar 1	14.34	−13	−14	−8	2	−*8*	−9	−6	−4	−12	−*8*
TR Bucking Bar 2	21.24	−3	0	−1	8	*1*	4	4	4	−3	*2*
Paving Breaker	52.26	0	−4	−1	−1	−*2*	4	3	5	−2	*3*
Clay Spade	27.25	−4	−2	−2	−9	−*4*	1	0	2	−9	−*2*
Chipping Hammer B	12.16	−4	−3	1	18	*3*	3	4	4	−2	*2*
Chipping Hammer A	10.95	−5	0	0	14	*2*	5	0	5	−1	*2*
Impact Drill	9.08	−10	−10	−6	16	−*3*	−9	−7	−4	11	−*2*
Rotary Hammer	18.91	−8	−5	−1	2	−*3*	3	0	4	−4	*1*
Rock Drill	11.70	0	−2	1	7	*2*	8	7	7	−1	*5*
Stone Hammer – Chisel	19.31	−20	−17	−8	8	−*9*	−12	−10	−5	−7	−*9*
Stone Hammer – Handle	21.31	−2	0	−5	4	−*1*	2	1	0	−5	−*1*
3kg Impact Drill	11.71	−6	−10	−4	18	−*1*	−1	0	1	−4	−*1*
6kg Impact Drill	9.44	−7	−5	−3	12	−*1*	4	2	4	−3	*2*
Needle Scaler	11.89	−14	−12	−6	−4	−*9*	−16	−10	−6	−17	−*12*
Scabbler	12.80	7	6	6	−2	*4*	9	7	8	−1	*6*
PC Sander	9.90	−6	−5	−4	−7	−*6*	−6	−6	−5	−19	−*9*
P Air Sander	2.69	−6	−3	−1	22	*3*	−3	−2	0	5	*0*
Belt Sander	3.80	−5	−1	0	24	*5*	−3	−1	0	17	*3*
BD Sander	17.80	−6	−3	−7	−16	−*8*	−4	−2	−7	−20	−*8*
Orbit Sander	4.79	−7	−7	−3	−6	−*6*	−2	−4	0	−12	−*5*
Pavement Cutting Saw	12.12	−6	−4	−6	−12	−*7*	−4	−3	−5	−16	−*7*
Miter Saw	5.47	−6	−2	−4	2	−*3*	−2	1	−4	−11	−*4*
Jig Saw	6.77	−10	−9	−4	6	−*4*	−4	−4	0	−10	−*5*
Circular Saw	6.11	−6	0	−2	13	*1*	−2	0	−3	−5	−*3*
Reciprocating Saw	5.78	−8	−7	−5	14	−*2*	2	1	2	−1	*1*
Chain Saw	9.93	6	4	4	−6	*2*	9	5	7	−4	*4*
Multi-use Tool	11.84	−8	−3	−1	17	*1*	−8	−3	−1	16	*1*
Golf Club Head-Grinding	2.59	−5	−7	−3	12	−*1*	6	4	5	−1	*4*
Vertical Grinder ship yard	11.33	−11	−17	−9	15	−*6*	−6	−10	−4	0	−*5*
7 In Grinder	6.10	−7	−4	−3	14	*0*	3	2	4	−2	*2*
4.5 In Grinder	11.99	−4	−7	−5	−5	−*5*	−1	−3	−1	−13	−*5*
Electric Angular Grinder	9.99	−4	−7	−2	2	−*3*	1	−3	2	−9	−*2*
Angular Grinder	5.47	−6	0	−1	13	*2*	−1	0	−1	−3	−*1*
Die Grinder	1.69	−6	−2	0	19	*3*	0	0	1	0	*0*
Pencil Grinder	1.57	−5	−14	−8	10	−*4*	1	0	1	0	*1*
Hedge Trimmer	13.48	−4	−1	−3	4	−*1*	3	3	2	−6	*1*
Strimmer	7.11	−6	−6	−5	7	−*3*	−3	−2	−4	−11	−*5*
Trimmer	13.48	−4	−1	−3	4	−*1*	3	3	2	−6	*1*
Angular Nutrunner	2.88	−4	−1	−2	6	*0*	2	2	3	−2	*1*
Straight Nutrunner 1	2.42	−5	−3	−4	5	−*2*	1	0	1	−1	*0*
Straight Nutrunner 2	2.17	−7	−5	−4	2	−*4*	0	0	1	−3	−*1*
Straight Nutrunner 3	2.93	−8	−7	−4	2	−*4*	0	−1	1	−5	−*1*
Angular Nutrunner	16.70	−5	−1	−2	12	*1*	0	1	1	−1	*0*
Pistol Nutrunner	2.57	−6	−8	−4	11	−*2*	2	1	2	−1	*1*
Pistol Screw Gunn 1	4.19	−4	−4	−1	12	*1*	2	1	3	−1	*1*
Pistol Screw Gunn 2	5.55	−2	−3	0	2	−*1*	2	1	3	−1	*1*
Pistol Screw Gunn 3	2.99	−4	−2	0	6	*0*	2	2	3	−2	*1*
*Mean reduction for glove/tool combinations*		−*6*	−*5*	−*3*	*6*	−*2*	*0*	*0*	*1*	−*4*	−*1*

Negative = Increase; Positive = Reduction of Vibration.

**Table 5:** Effectiveness of the gloves on tools/workpieces with the dominant vibration in shear direction or with the vibration distributed over the three orthogonal directions (hand-held workpiece). Tool accelerations in m/s^2^; Transmissibilities are unit less

	Unweighted			Weighted			Unweighted			Weighted		
	Ay	Axz	At	Ay	Axz	At	Ay	Axz	At	Ay	Axz	At
	Stone hammer – chisel						Stone hammer – handle					
m/s^2^	217.55	120.82	248.84	17.35	8.46	19.31	60.01	173.69	183.77	9.90	18.88	21.31
Fingertip	Ty	Txz	Txzy	Ty	Txz	Txzy	Ty	Txz	Txzy	Ty	Txz	Txzy
Gel	1.25	0.98	1.19	1.17	1.03	1.14	1.03	1.02	1.02	1.11	1.05	1.06
Air Bladder	1.46	1.04	1.38	1.18	1.06	1.16	1.13	1.07	1.07	1.04	1.06	1.06
Air Bubble	1.24	0.89	1.17	1.12	1.00	1.10	1.09	0.97	0.99	1.07	1.05	1.06
Neoprene	0.95	0.64	0.89	1.10	1.02	1.09	1.06	0.83	0.85	1.18	1.10	1.12
Proximal												
Gel	1.23	1.13	1.21	1.13	0.98	1.10	1.19	1.02	1.04	1.06	0.86	0.91
Air Bladder	1.01	0.97	1.00	1.08	0.92	1.05	1.07	0.91	0.93	1.11	0.86	0.92
Air Bubble	0.97	1.12	1.01	1.01	1.01	1.01	1.02	1.13	1.12	1.06	0.94	0.97
Neoprene	0.95	1.03	0.97	1.06	1.01	1.05	1.07	1.09	1.09	1.14	0.98	1.01
	Golf club head – grinding process						Needle scaler					
m/s^2^	14.77	19.89	24.79	1.04	2.37	2.59	73.16	41.22	83.97	11.38	3.45	11.89
Fingertip	Ty	Txz	Txzy	Ty	Txz	Txzy	Ty	Txz	Txzy	Ty	Txz	Txzy
Gel	1.17	0.99	1.05	1.11	1.06	1.07	1.16	1.01	1.13	1.20	1.04	1.19
Air Bladder	1.63	1.04	1.27	1.21	1.06	1.09	1.27	1.06	1.22	1.07	1.06	1.07
Air Bubble	1.28	0.95	1.07	1.12	1.11	1.12	1.12	0.93	1.07	1.06	1.00	1.06
Neoprene	0.94	0.80	0.86	1.08	1.18	1.16	1.13	0.74	1.05	1.27	1.04	1.25
Proximal												
Gel	1.14	1.05	1.07	1.01	0.78	0.82	1.18	1.09	1.16	1.14	0.98	1.13
Air Bladder	0.89	0.92	0.91	1.03	0.80	0.84	1.08	0.94	1.05	1.16	0.94	1.14
Air Bubble	1.02	1.01	1.01	1.01	0.75	0.79	1.04	1.13	1.06	1.07	1.03	1.06
Neoprene	0.95	0.94	0.94	1.03	0.83	0.86	1.04	1.07	1.05	1.10	1.03	1.10
	Steel Bucking Bar B						Tungsten Bucking Bar E					
m/s^2^	79.94	35.33	87.40	12.07	8.67	14.86	27.09	13.42	30.23	5.17	3.96	6.51
Fingertip	Ty	Txz	Txzy	Ty	Txz	Txzy	Ty	Txz	Txzy	Ty	Txz	Txzy
Gel	1.14	1.02	1.12	1.08	1.04	1.06	1.14	1.02	1.11	1.06	1.04	1.05
Air Bladder	1.47	1.05	1.41	1.05	1.03	1.04	1.41	1.05	1.34	1.03	1.03	1.03
Air Bubble	1.21	0.98	1.18	1.05	1.05	1.05	1.19	1.00	1.16	1.03	1.05	1.04
Neoprene	0.98	0.96	0.98	1.12	1.12	1.12	1.01	1.02	1.01	1.09	1.13	1.11
Proximal												
Gel	1.16	1.03	1.14	1.00	0.90	0.97	1.16	0.99	1.13	1.00	0.89	0.96
Air Bladder	0.96	0.94	0.96	1.06	0.92	1.02	0.99	0.93	0.98	1.04	0.91	1.00
Air Bubble	1.01	1.05	1.01	0.98	0.87	0.94	1.00	1.00	1.00	0.98	0.86	0.94
Neoprene	0.98	1.03	0.99	1.05	0.92	1.01	0.99	1.00	1.00	1.04	0.91	0.99
